# Screening and identification of multiple abiotic stress responsive candidate genes based on hybrid-sequencing in *Vicia sativa*

**DOI:** 10.1016/j.heliyon.2023.e13536

**Published:** 2023-02-04

**Authors:** Jia Wei, Bo Luo, Shiyi Kong, Wenxian Liu, Chuanjie Zhang, Zhenwu Wei, Xueyang Min

**Affiliations:** a College of Animal Science and Technology, Yangzhou University, Yangzhou, Jiangsu Province, 225009, People’s Republic of China; b State Key Laboratory of Grassland Agro-ecosystems, College of Pastoral Agriculture Science and Technology, Lanzhou University, Lanzhou, 730000, People’s Republic of China

**Keywords:** *Vicia sativa* L, Hybrid-sequencing, Gene expression atlas, Multiple abiotic stress, Candidate genes

## Abstract

Common vetch is an important leguminous forage for both livestock fodder and green manure and has a tremendous latent capacity in a sustainable agroecosystem. In the present study, a comprehensive transcriptome analysis of the aboveground leaves and underground roots of common vetch under multiple abiotic stress treatments, including NaCl, drought, cold, and cold drought, was performed using hybrid-sequencing technology, i. e. single-molecule real-time sequencing technology (SMRT) and supplemented by next-generation sequencing (NGS) technology. A total of 485,038 reads of insert (ROIs) with a mean length of 2606 bp and 228,261 full-length nonchimeric (FLNC) reads were generated. After deduplication, 39,709 transcripts were generated. Of these transcripts, we identified 1059 alternative splicing (AS) events, 17,227 simple sequence repeats (SSRs), and 1647 putative transcription factors (TFs). Furthermore, 640 candidates long noncoding RNAs (lncRNAs) and 28,256 complete coding sequences (CDSs) were identified. In gene annotation analyses, a total of 38,826 transcripts (97.78%) were annotated in eight public databases. Finally, seven multiple abiotic stress-responsive candidate genes were obtained through gene expression, annotation information, and protein-protein interaction (PPI) networks. Our research not only enriched the structural information of FL transcripts in common vetch, but also provided useful information for exploring the molecular mechanism of multiple abiotic stress tolerance between aboveground and underground tissues in common vetch and related legumes.

## 1 Introduction

Plants cannot move, so abiotic factors are the fundamental components that determine plant distribution and productivity. In the natural environment, plants are constantly challenged by adverse abiotic environmental conditions (e.g., extreme temperatures, drought, nutrient deficiencies, and soil salinization). These stressors greatly limit plant growth and development, negatively impacting crop productivity [1]. Previous estimates indicate that the adverse environment has resulted in a loss of nearly 70% of the overall crop yield, implying that the average yield is generally 30% of the theoretical maximum yield potential [2]. It follows that the impact of abiotic stresses on plant growth throughout their life cycle is not only a basic biological issue, but also the crux of the matter in achieving ecological environment, agricultural sustainability, and food security with an ever-increasing world population [3,4]. Among various unfavorable environmental conditions, drought, salt, and temperature stresses are three main abiotic factors and always occur simultaneously, so it is important to understand how common and tissue-specific response pathways interact. Thus, understanding plant responses to multiple abiotic stresses among aboveground and underground tissues at the molecular level will be necessary to identify key molecular targets for further molecular breeding.

Recent progress in NGS technology, a massively parallel short-read technology [5], provides a cost-effective and efficient way to acquire rich transcriptome data from multiple tissue samples and organism types [6,7]. In recent years, the application of NGS technology in many in-depth studies of common vetch has been well documented. Liu et al. used NGS technology, combined with quantification analysis, to develop the first transcriptome expression analysis for a single flower organ from common vetch. By comparing the transcripts enrichment percentage and gene expression patterns in six flower organs of common vetch and *Arabidopsis thaliana*, they provided substantial insight into the evolution and development of zygomorphic flowers [8]. NGS technology was also used to assess the overall changes in the transcriptome of the shattering-susceptible and shattering-resistant ventral sutures of common vetch accessions. Twenty-two differentially expressed genes have been identified that encode cell wall modifications and hydrolytic enzymes related to pod rupture, and these genes were robustly expressed in shattering-susceptible accessions and provided a new perspective for understanding the mechanism of the vetch pod shattering [9]. Compared with the complex and huge whole genome, the gene fragments of a single organ or tissue of common vetch are smaller, which has obvious advantages in sequencing cost and quality. Albeit the high-throughput and high-precision of NGS has its unique advantages, NGS has a major limitation: the length of each read is relatively short, and partial gene deletions and assembly errors occur from time to time, which will affect the acquisition of the FL transcriptome and genome-wide information of the species [10].

SMRT, also known as the third-generation sequencing [11], offers much longer read lengths and faster runs than NGS, but is hindered by lower throughput data and higher error rates. Since the advantages of SMRT and NGS are complementary, hybrid-sequencing technology has become a new research upsurge. This technology overcomes the problems of short splicing and incomplete information of transcripts of species without reference genome information. At the same time, it can also use the NGS data to carry out transcript specific expression analysis to obtain more comprehensive annotation information [12,13]. In general, the technology involves using high-throughput and high-accuracy short-read data to correct errors in the long reads [12]. To our knowledge, several economically valued plants are being sequenced, and the understanding of the important characteristics of abiotic stress resistance is being carried out using hybrid-sequencing technology. For instance, Zhao et al. (2021) utilized the PacBio and BGISEQ-500 RNA-seq platforms to study the molecular mechanism of *Arachis glabrata* in response to biotic and abiotic stresses [14]. Recently, Wang et al. (2021) used hybridization sequencing technology to compare and analyze gene expression patterns of linseed varieties with different drought resistance under drought and repeated drought conditions, indicating that it is suitable for revealing the drought resistance mechanism of linseed [15].

Common vetch (*Vicia sativa* L.) is an important annual, self-pollinated, and diploid leguminous forage [16,17]. It is a kind of multi-functional forage that plays a unique role at different growth stages. Its roots can be symbiotic with rhizobia for nitrogen fixation and can be used as high-efficiency green manure after returning to the field; the nutrient growth part after harvest can be used for livestock fodder, silage, or hay [18,19]; the seed can be safely used as a protein-rich feed additive for ruminants and is a sustainable source of plant protein [19]. Notably, increasing evidence indicates that, compared with other annual or perennial legume forages, common vetch has strong adaptability to changeable climate patterns [[20], [21], [22]]. Thereupon, its stress resistance has garnered extensive attention. In order to better understand how common vetch adapts to abiotic stresses, it is necessary to study the response of common vetch to multiple individual and combined stresses in detail. The main objective of this study was to characterize FL transcripts from aboveground leaves and underground roots of common vetch under different abiotic stress treatments using hybrid-sequencing technology to unveil the complex transcriptome. Based on the obtained transcripts, CDSs prediction, AS events identification, lncRNAs and their targeted transcripts prediction, SSR analysis, TFs prediction, and transcripts functional annotation were performed. Furthermore, we predicted the multiple abiotic stress-related core genes and their potential functional pathways by screening differentially expressed genes (DEGs) and exploiting transcripts functional annotation data. Our research will serve as an important complement to the whole genome structure in common vetch, contribute to the current body of knowledge on the genome, and provide a clearer background for the study of stress resistance in common vetch.

## 2 Materials and methods

### 2.1 Plant material collection

Firstly, the cultivar “Lanjian No. 1” seeds were sterilized in a 1% sodium hypochlorite solution for 5 min and rinsed with distilled water six times, after which they were placed on Petri dishes with two layers of filter paper that were moistened with distilled water and allowed to germinate at 25 °C. After 4 days, 25 seedlings with similar growth were selected and sown in 60-well plates, respectively. Then hydroponics was conducted in half-strength Murashige and Skoog (1/2 MS) nutrient solution with a pH of 5.8. The seedlings were grown in a controlled growth chamber maintained at 22 °C with a relative humidity of 80% under long-day conditions (16 h light/8 h dark) for one week. Combining our previous phenotypic and physiological results with Wang et al.’s findings [[23], [24], [25]], and in order to avoid the influence of different photoperiods on experimental materials, we chose 24 h (a full photoperiod) to carry out abiotic stress treatment [23,25]. And then the seedlings were evenly divided into 5 groups for different treatments. In order to screen the appropriate reagent concentration and environmental temperature for each stress treatment, extensive preliminary experiments have been carried out before the implementation of this experiment. One group was randomly selected and cultivated under a normal condition (25 °C) as a control; The remaining groups were treated with 150 mM NaCl, 20% polyethylene glycol (PEG), 4 °C, and cold drought (4 °C + 20% PEG) combined stress for 24 h, respectively. Four biological replicas were made in each group, and the aboveground (leaves) and underground (roots) tissues were harvested after 24 h. Each sample was collected from four single uniform seedlings of the same treatment, with three biological replicates. All collected samples were frozen in liquid nitrogen and stored at −80 °C for RNA extraction.

### 2.2 RNA isolation and extraction

According to the manufacturer’s instructions, total RNA was extracted using Trizol (Invitrogen, USA) and RNeasy Plus Mini Kit (Agilent Technologies, CA, USA). A total of 33 samples [2 tissues (aboveground leaves and belowground roots) × 5 treatments (control, NaCl/24 h, 20% PEG/24 h, 4 °C/24 h and 4 °C + PEG/24 h) × 3 biological replications, and stem tissue (control) × 3 biological replications] were used for transcriptome analysis. RNA degradation and contamination were assessed using 1% agarose gels. The quality and quantity of the RNA have been assessed in Agilent 2100 Bioanalyzer (Agilent Technologies, USA) and RNA Nano 6000 Assay Kit. For PacBio isoform sequencing (Iso-Seq) (Pacific Bioscience, Menlo Park, USA), equal amounts of total RNA with an RNA integrity number ≥ 7.0 and a 28S/18S ratio ≥ 1.0 from each sample pooling were carried out.

### 2.3 PacBio library construction and sequencing

Equivalent amounts of total RNA from 33 samples were combined to construct a representative sample for sequencing. Using SMARter™ PCR cDNA synthesis kit synthesized the FL cDNA of mRNA. Initially, the FL cDNA fragments were screened by the BluePippin size selection system, and cDNA libraries of different sizes were constructed. Hereafter, the FL cDNA was amplified and screened by PCR again, the end of the FL cDNA was repaired, and the SMRT dumbbell connector was connected for exonuclease digestion. Finally, BluePippin was used for secondary screening, and three libraries were then generated. Library size and quantification were assessed on the Agilent Bioanalyzer 2100 system and Qubit 2.0, respectively. After the library test was qualified, the third-generation transcriptome sequencing was performed by PacBio RS II. Seven SMRT cells were conducted in this study: three, two, and three SMRT cells sequenced three libraries with size ranges of 1–2, 2–3, and 3–6 KB, respectively.

The ToFu pipeline parameters with full pass ≥ 0, prediction accuracy > 0.75, and sequence length ≥ 300 bp were used to screen the sequenced PacBio ROIs. Next, non-full-length (NFL) and FLNC transcripts were determined according to whether there were 3′ primers, 5′ primers, and polyA in the ROIs. The consensus isoforms were obtained by using the iterative isoform-clustering program (ICE), the NFL sequences were clustered by the Quiver algorithm, and the consistent sequences were corrected to obtain high-quality and low-quality isoforms, respectively. The data from second-generation transcriptome sequencing were used to correct the low-quality isoforms, and CD-HIT (identity > 0.99) deleted redundancy to obtain the transcripts [26].

### 2.4 cDNA library construction and sequencing

Eukaryotic mRNA was enriched with magnetic beads with Oligo (dT), and the mRNA was randomly interrupted by adding a fragmentation buffer. The first cDNA strand was synthesized with six base random primers (random hexamers) using mRNA as a template [7], and then the second cDNA strand was synthesized by adding buffer, dNTPs, RNase H, and DNA Polymerase I. The cDNA was purified by AMPure XP beads, and the purified double-stranded cDNA was subjected to terminal repair, added a tail, and connected to the sequencing adapter. Then selected the fragment size with AMPure XP beads, and finally enriched the cDNA library by PCR. The library test was the same as the PacBio library mentioned above. Index-coded samples were clustered following the manufacturer’s protocol. Once samples were clustered, libraries were then submitted on an Illumina HiSeq X Ten platform (Illumina, San Diego, USA) for sequencing to generate paired-end reads. Finally, each sample yielded more than 6 Gb of clean data.

All of the raw data in the fastq format was processed using internal Perl scripts before assembly. At this stage, clean data were obtained by deleting three kinds of reads: one with adaptors, another of low-quality, and a third with more than 10% unknown bases. Calculated the parameter Q30 (1 error in 1000 bases) and GC content at the same time to evaluate the quality of these clean data [27].

### 2.5 Structural analysis of FL transcriptome

TransDecoder (version 3.0.0) software identified reliable CDSs from transcripts based on the length of the open reading frame (ORF), Log-likelihood Score and the alignment of amino acid sequences with protein domain sequences in Pfam database.

Through IsoSeq_AS_*de*_*novo* script, the AS events of each sample were obtained. The prediction principle of this method was to use BLAST software (version 2.2.26) to compare the sequences in pairs. The alignment results met the following conditions: the lengths of both sequences were greater than 1000 bp, and contained two HSPs (High-scoring Segment Pairs) in the alignment; The AS gap was greater than 100 bp and at least 100 bp away from the end of 3′/5′; Allowing an overlap of 5 bp of the all alternative transcripts, then were considered as candidate AS events [28].

The transcripts were predicted by four coding potential analysis algorithms: CPC (Coding Potential Calculator), CNCI (Coding-Non-Coding Index), CPAT (Coding Potential Assessment Tool), and Pfam (Protein family). The overlapping data of the four analysis results were used for subsequent lncRNAs analysis. Based on the complementary pairing of lncRNA and mRNA bases, the LncTar prediction tool was used to predict the targeted transcripts of each lncRNA [29].

More than 500 bp transcripts were screened from the new transcripts and analyzed by SSRs with MISA (MIcroSAtellite identification tool). The repeat sequence motifs included perfect mono-, di-, tri-, tetra-, penta-, and hexa-nucleotides with a minimum repeat number of ten, six, five, five, five, and five, respectively. Batch Primer 3, an online web tool, was used to design SSR primer pairs from the flanking sequences of the identified microsatellite motifs; three pairs of primers were designed for each SSR marker [30,31].

### 2.6 Functional annotation of transcripts and TFs prediction

To obtain the annotation information of the transcripts, BLAST software (E-value ≤ 10^−5^) was used to compare the obtained transcript sequence with eight different protein and nucleotide databases, including NR (NCBI non-redundant proteins), Swiss-Prot (a manually annotated, non-redundant protein database), eggNOG (evolutionary genealogy of genes: non-supervised orthologous groups), COG (Clusters of Orthologous Groups), KOG (euKaryotic Orthologous Groups), Pfam (a database of conserved Protein families or domains), GO (Gene Ontology), and KEGG (Kyoto Encyclopedia of Genes and Genomes) [10,31]. Potential TFs were predicted from the Plant Transcription Factor Database (PlantTFDB) [27].

### 2.7 DEGs analysis and construction of PPI networks

The transcript expression levels were calculated by the fragments per kilobase of transcript per million mapped reads (FPKM) method for each sample. Thereafter, by comparing the treated samples and control samples in pairs, set the thresholds at: false discovery rate (FDR) < 0.01 and |log_2_ (Fold Change)| ≥ 2 to determine the DEGs. The DEGs of the leaves and roots that responded to all stresses were selected for principal component analysis (PCA), respectively. The Blast program was used to screen the homologous genes of seven core genes in common vetch from *Medicago sativa*, *A. thaliana* and *Glycine max*, and analyze their expression patterns under different abiotic stresses. *M. sativa* data was quoted from the literature of Luo et al. [32], *A. thaliana* data was from http://bar.utoronto.ca/efp/cgi-bin/efpWeb.cgi, and *G. max* data was downloaded from http://ipf.sustech.edu.cn/pub/plantrna/.

The CDSs of the selected DEGs were interrogated using the STRING v11.5 (Search Tool for the Retrieval of Interacting Genes/Proteins) database for the PPI networks analysis. The PPI networks were constructed using *A. thaliana* as a reference, with predicted functional partners obtained from a database. Parameter setting: the meaning of edges was set to line color to indicate the type of interaction evidence, with seven active interaction sources selected by default; the interactions of selected genes were set at a medium confidence level (level of 0.400); and the maximum number of interactions shown by the first and the second shell was set to <10. The STRING resource is available online at http://string-db.org/.

## 3 Results

### 3.1 Transcripts analysis from SMRT sequencing

To obtain a representative common vetch FL transcriptome, three vegetative organs of common vetch, including roots, stems, and leaves, as well as root and leaf tissue samples under different stress treatments, including NaCl, drought, cold and cold drought stresses, were collected in equal quantities for SMRT sequencing. Three cDNA libraries with the sizes of 1–2 K, 2–3 K and 3–6 K were constructed and then performed by PacBio RS II. A total of 726,451 post-filter polymerase reads were obtained, resulting in 8,771,461 post-filter subreads with a mean N50 of 2666 bp and an average subread length of 2045 bp (Table S1). According to the condition that the min full passes were zero and the sequence accuracy was greater than 0.75, the ROI sequences were extracted from the raw sequence reads, and the total number of ROI sequences was 485,038 with a mean length of 2606 bp and a read quality of 0.92. After 56,760 short sequences (<300 bp) were removed, 199,115 bar NFL reads and 229,163 bar FL reads were distinguished. At the same time, 228,261 bar FLNC reads with an average length of 2144 bp were obtained (Table 1). By ICE analysis, a total of 79,879 consensus isoforms emerged, including 64,559 high-quality isoforms and 15,320 low-quality isoforms, and the 15,320 low-quality isoforms were corrected using 311.04 Gb second-generation clean data. CD-HIT eliminated the redundancy of high-quality isoforms and corrected the low-quality isoforms in each sample. Finally, 39,709 non-redundant, high-quality isoforms were obtained (Table S2).

**Table 1:** Statistics of SMRT sequencing data.

Terms	Amount
Number of reads of insert	485,038
Mean read length of insert (bp)	2606
Mean read quality of insert	0.92
Number of filtered short reads	56,760
Number of non-full length reads	199,115
Number of full-length reads	229,163
Number of full-length non-chimeric reads	228,261
Average full-length non-chimeric read length (bp)	2144
Full-length percentage (FL%)	47.25
Number of polished high-quality isoforms	64,559
Number of polished low-quality isoforms	15,320

### 3.2 CDSs prediction and AS events identification

**Fig. 1:**
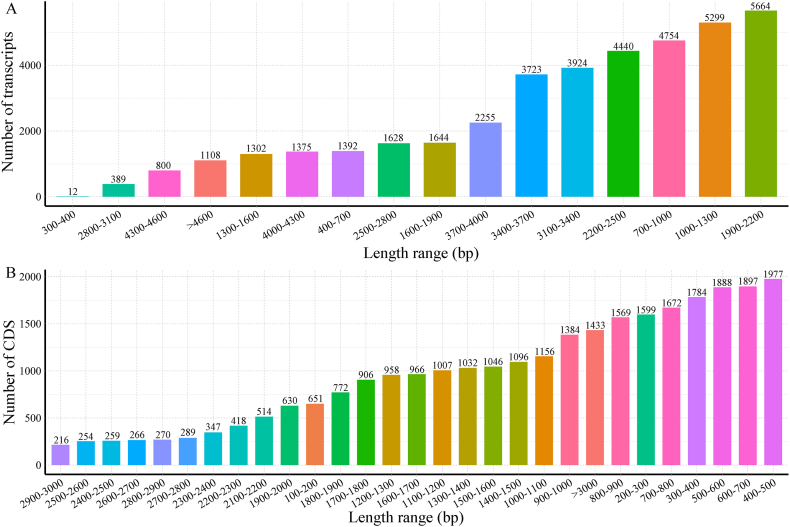
The length distribution of transcripts (A) and CDSs (B).

From 39,709 non-redundant high-quality isoforms, a total of 38,228 ORFs with a mean length of 1180.95 bp were predicted (Table 2), including 28,256 complete ORFs with start codons and stop codons, 204 3′ prime partial sequences with only the predicted stop codons, 9730 5′ prime partial sequences with only the start codons predicted, and 38 internal sequences, that is, the type of CDSs for which neither the start codons nor the stop codons were predicted. The minimum length of CDSs was 150 bp, and the maximum length was 4800 bp. Next, the length distributions of transcripts (Fig. 1A) and complete CDSs were investigated (Fig. 1B). PacBio SMRT sequencing identified events by directly comparing different subtypes of the same gene. Here, 1059 AS events were predicted (Table S3).

**Table 2:** Statistics of transcripts and CDSs length.

	Transcripts	CDSs	Complete CDSs
Number of sequences	39,709	38,228	28,256
Total length (bp)	94,604,849	45,145,464	33,699,720
Minimum length (bp)	304	150	150
Maximum length (bp)	14,891	4800	4674
Mean length (bp)	2382.45	1180.95	1192.65
N50 (bp)	2189	942	978

### 3.3 Prediction of LncRNAs and their target transcripts

The transcripts were screened for coding potential by four analysis algorithms, including CPC, CNCI, CPAT, and Pfam, to judge whether they have coding potential, so as to infer whether the transcripts are candidate lncRNAs. A total of 9745 potential lncRNAs were predicted by the four kinds of analysis algorithms. Altogether, 1597, 1798, 5796 and 5937 lncRNAs were identified in CPC, CNCI, CPAT and Pfam algorithms, respectively (Table S4). By filtering out transcripts with a length of less than 300 bp, 640 transcripts were considered candidate lncRNAs, which had no coding potential in all four methods mentioned above (Figure S1). At the same time, 338 target transcripts corresponding to lncRNAs were predicted (Table S5).

### 3.4 SSRs analysis

**Fig. 2:**
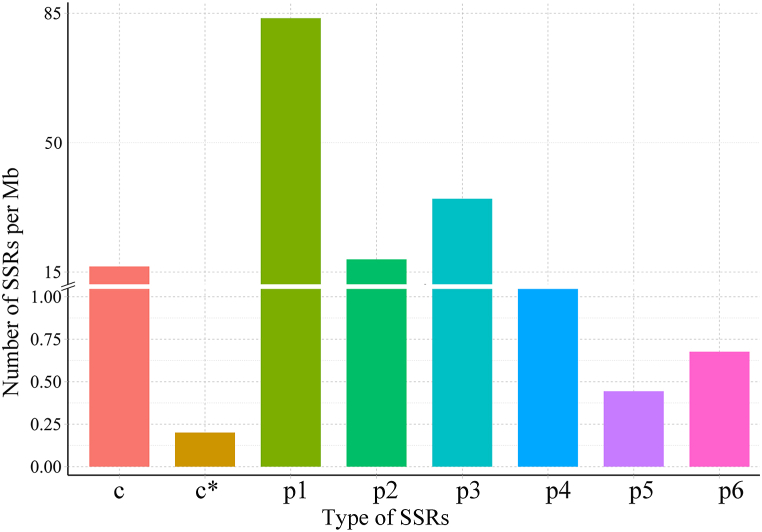
Statistics and characteristics of the SSRs. X-axis represented the SSR types and Y-axis was the number of SSRs contained in transcripts per Mb length.

Among the 39,709 obtained transcripts, 63 transcripts with a length of less than 500 bp were deleted, and the remaining 39,646 transcripts were used for SSRs analysis. A total of 17,227 SSRs were identified in 11,183 sequences and 3228 transcripts that contained more than one SSR. Furthermore, 2451 (14.23%) SSRs were presented in the form of compound formations. Not only that, predominant SSRs were mono-nucleotide repeats (7911, 45.92%), followed by tri-nucleotide repeats (3293, 19.12%), di-nucleotide repeats (1743, 10.12%), and then tetra-nucleotide repeats (143, 0.83%), the number of penta- (42, 0.24%) and hexa-nucleotide repeats (64, 0.37%) were both not over one hundred (Table 3). It could also be seen from the SSR types density map contained in each 1 Mb long transcript that most of these SSRs have mono-nucleotide repeats (Fig. 2). The 2451 SSRs in the form of the compound were not considered, according to the principle of designing three pairs of primers at both ends of each SSR, 34,956 pairs of primers were successfully designed for the remaining 14,776 SSRs, and 3124 SSRs failed to design primers at both ends, most of them were SSRs close to the front or tail of the sequence (Table S6).

**Table 3:** Statistical table of SSRs identified in the *Vicia sativa* transcriptome.

Searching item	Numbers
Total number of sequences examined	39,646
Total size of examined sequences (bp)	94,576,743
Total number of identified SSRs	17,227
Number of SSR containing sequences	11,183
Number of sequences containing more than one SSR	3228
Number of SSRs present in compound formation	2451
Mono-nucleotide	7911
Di-nucleotide	1743
Tri-nucleotide	3293
Tetra-nucleotide	143
Penta-nucleotide	42
Hexa-nucleotide	64

### 3.5 TFs prediction

**Fig. 3:**
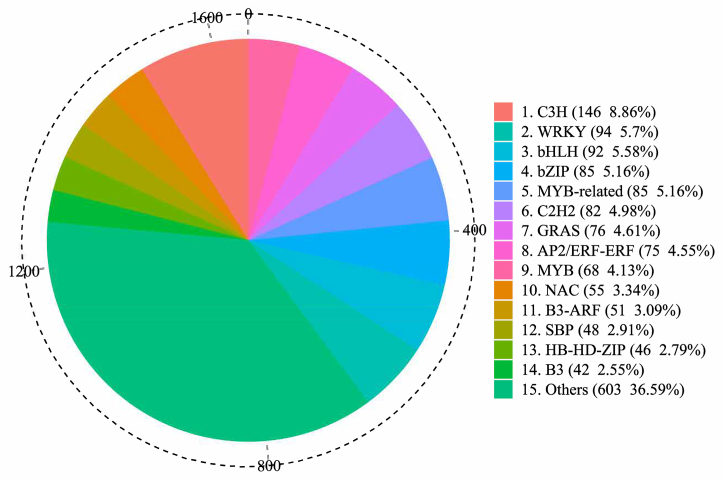
Distribution of TF types in the *Vicia sativa* transcriptome.

Totally, 1647 putative TFs were identified in this study, and TFs presented in common vetch were classified into 64 TF families (Table S7). In detail, the TFs in common vetch transcriptome were mainly assigned to the following families: Cysteine3Histidine (C3H) (146, 8.86%), WRKY (94, 5.71%), basic helix-loop-helix (bHLH) (92, 5.59%), basic leucine zipper (bZIP) (85, 5.16%), MYB-related (85, 5.16%), C2H2 (82, 4.98%), GRAS (76, 4.61%), AP2/ERF-ERF (75, 4.55%), MYB (68, 4.13%), NAC (55, 3.34%), B3-ARF (51, 3.10%), SBP (48, 2.91%), HB-HD-ZIP (46, 2.79%) and B3 (42, 2.55%) families (Fig. 3).

### 3.6 Transcript functional annotation

In this study, all 39,709 non-redundant transcripts were functionally annotated by searching and comparing to the GO, KEGG, KOG, Pfam, Swiss-Prot, COG, eggNOG, and NR databases. Therein, 38,826 transcripts (97.78%) were annotated, and the remaining 883 transcripts were not hit in any of the above databases. In the subdivision of the transcript annotation, 25,188 (63.43%), 17,470 (44.00%), 24,917 (62.75%), 32,343 (81.45%), 29,965 (75.46%), 17,154 (43.20%), 37,643 (94.80%), and 38,665 (97.37%) transcripts were annotated into the GO, KEGG, KOG, Pfam, Swiss-Prot, COG, eggNOG, and NR databases, respectively (Table S8).

### 3.7 GO annotation and enrichment analysis

The gene functions of transcripts were classified by GO annotation. In general, Go analysis showed that 25,188 enriched transcripts were divided into three main branches, which could be categorized into 52 sub-terms, including 20, 17, and 15 sub-terms under the branch of “biological process”, “cell component” and “molecular functional” terms, respectively (Figure S2). For “cellular component” classification, the highly abundant sub-terms were “cell part” (10,079, 40.02%), “cell” (10,064, 39.96%), and “organelle” (7456, 29.60%). The major subclass of “molecular function” terms were “binding” (13,796, 54.77%) and “catalytic activity” (13,663, 54.24%). In the “biological process” classification, with most representation were “metabolic process” (17,291, 68.65%), “cellular process” (15,029, 59.67%), and “single-organism process” (11,757, 46.68%).

### 3.8 COG classification

To further understand the classification of potential gene products of transcripts in common vetch, all consensus sequences were aligned to the COG database to predict and classify possible functions. The results indicated that 17,154 transcripts were annotated, of which 3426, 2272, 4727, and 3024 transcripts were only involved in four major functional categories: “cellular processes and signaling”, “information storage and processing”, “metabolism” and “poorly characterized”, respectively. There was a one-to-one correspondence between those transcripts and the function classes, while the functional classes of the remaining 3705 transcripts were not unique (Table S9). Further subdivided, 17,154 transcripts were assigned to 24 function classes. The largest class was “general function prediction only” (4916, 19.26%), followed by “transduction” (2661, 10.43%), another functional cluster with a percentage of transcripts greater than 10.00% was “replication, recombination, and repair” (2632, 10.31%). Based on the statistical chart, the proportion of transcripts in the four functional clusters was less than 1.00% (Figure S3).

### 3.9 KEGG pathway analysis

According to the results of searching and classifying transcripts in KEGG pathway database, we could roughly determine the biological functional pathways involved in common vetch. The results of transcripts enriched in KEGG pathways showed that a total of 17,470 transcripts were assigned to 127 KEGG functional pathways (Table S10). Among these pathways, “carbon metabolism” (795, 4.55%) ranked first, then came “ribosome” (650, 3.72%) and “biosynthesis of amino acids” (578, 3.31%), the “plant hormone signal translation” (524, 3.00%) and “starch and sucrose metabolism” (518, 2.97%) pathways had a similar number of transcripts, the number of transcripts of remaining KEGG pathways was less than 500 (Table 4).

**Table 4:** Statistics of the topmost 15 mapped pathways annotated by the KEGG database.

Serial number	Name of KEGG pathway	Pathway ID	Number of transcripts
1	Carbon metabolism	ko01200	795
2	Ribosome	ko03010	650
3	Biosynthesis of amino acids	ko01230	578
4	Plant hormone signal transduction	ko04075	524
5	Starch and sucrose metabolism	ko00500	518
6	Spliceosome	ko03040	498
7	Protein processing in endoplasmic reticulum	ko04141	486
8	RNA transport	ko03013	415
9	Oxidative phosphorylation	ko00190	383
10	Endocytosis	ko04144	366
11	Plant-pathogen interaction	ko04626	364
12	mRNA surveillance pathway	ko03015	360
13	Ubiquitin mediated proteolysis	ko04120	333
14	Phenylpropanoid biosynthesis	ko00940	329
15	Purine metabolism	ko00230	326

### 3.10 Expression data and PPI networks analysis of co-responsive DEGs

**Fig. 4:**
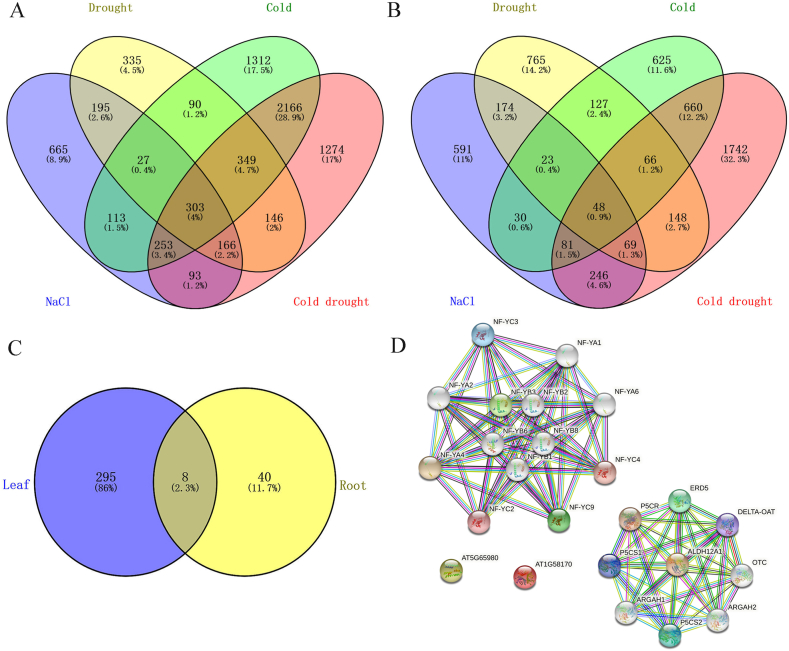
Venn diagrams of DEGs in leaves (A) and roots (B) under different stress treatments; (C) Venn diagrams of DEGs shared in the leaves and roots under different stress treatments; (D) A protein interactome network of 4 leaf-root co-responsive DEGs involved in stress response. Each protein node in the network internally displayed its 3D structural information, and each edge in the network represented a known or predicted interaction.

**Fig. 5:**
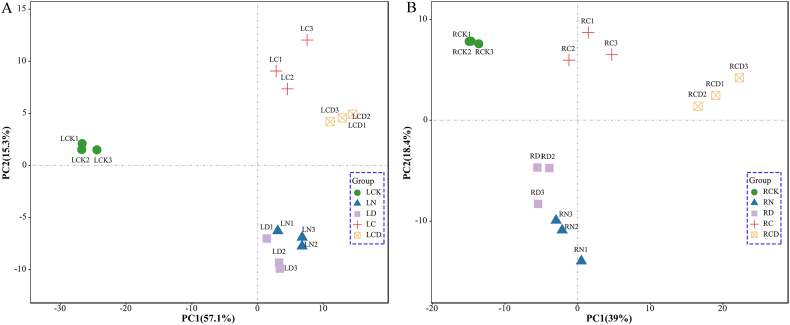
PCA plots of DEG in control and four abiotic stress treated samples of common vetch leaves (A) and roots (B). The shape of the same color represented the same stress treatment, and each treatment was three biological replicates. The percentage of total variance explained by each axis was provided in the figure. (For interpretation of the references to color in this figure legend, the reader is referred to the Web version of this article.)

A total of 665, 335, 1312 and 1274 DEGs were found in the leaves of common vetch, which responded only to single NaCl, drought, cold and cold drought stress, respectively, and 303 DEGs were found co-responsive in leaves under different stress treatments (Fig. 4A). Likewise, in roots, 591, 765, 625, and 1742 genes were specifically expressed in NaCl, drought, cold and cold drought stress, respectively, and 48 co-responsive DEGs were enriched in roots under different stress treatments (Fig. 4B). Subsequently, 303 co-responsive DEGs in leaves and 48 in roots under control and four abiotic stresses were analyzed by PCA to compare gene expression patterns (Fig. 5A and 5B). There was a certain degree of clustering within each treatment, indicating that the difference between experimental repetitions is small. There was an obvious separation and no overlap between the treatments, which indicated that the concentration of each stress treatment was correct and reasonable. It can be seen from the figure that the expression patterns of DEGs have changed to some extent after each treatment. DEGs of leaves and roots under salt stress and drought stress were likely to share a great similarity in gene expression, showing a certain degree of clustering and being far from the control. This may be because the essence of both treatments belongs to osmotic stress. There was a significant distance between the control sample and the cold drought treatment sample, indicating that the expression of DEGs had undergone a major shift.

**Fig. 6:**
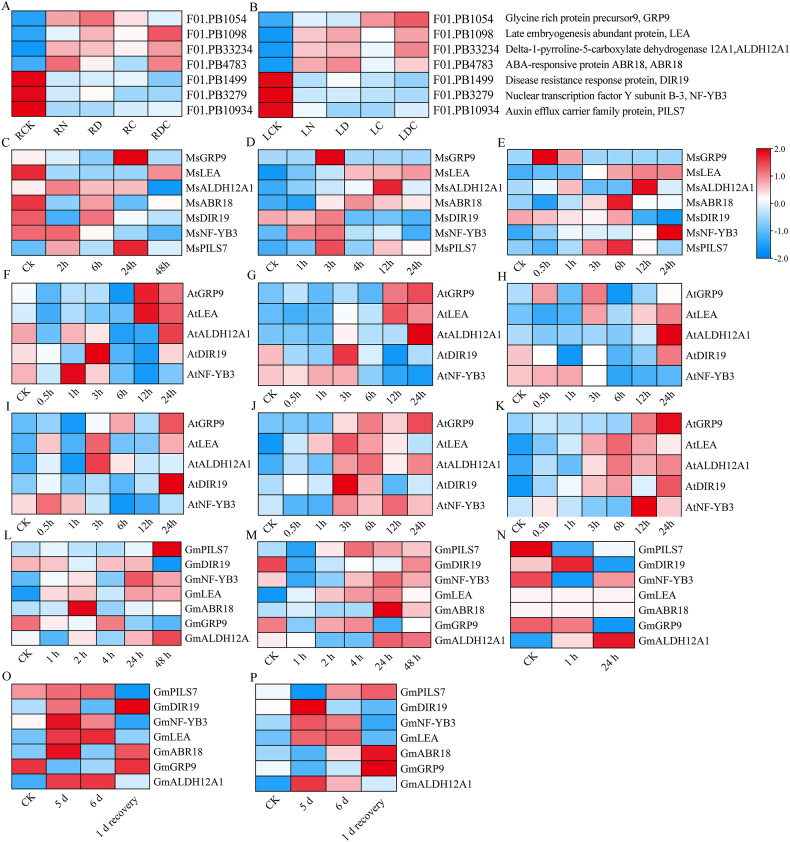
Heat map of expression of 7 co-responsive DEGs in leaves (A) and roots (B) of common vetch under different abiotic stress treatments. The expression levels of homologous genes in *M. sativa* roots exposed to low temperature stress (C), drought stress (D) and salt stress (E); Expression levels of homologous genes in *A. thaliana* shoots and roots exposed to low temperature stress (F, I), osmotic stress (G, J) and salt stress (H, K), respectively; The homologous genes expression levels in *G. max* leaves and roots exposed to salt stress (L, M) and drought stress (O, P) and *G. max* leaves exposed to low temperature stress (N), respectively. The color scale on the right represented the change from low expression (blue) to high expression (red) in the Log_2_ (FPKM) value of DEGs calculated after data normalization processing. (For interpretation of the references to color in this figure legend, the reader is referred to the Web version of this article.)

Through the Venn diagram, we obtained eight genes shared in leaves and roots that responded to all stress treatments (Fig. 4C). In the multiple sequence alignment analysis, we found “*F01.PB1098*” and “*F01.PB5441*” are highly similar, and “*F01.PB1098*” completely contains the sequence of “*F01.PB5441*”, so we keep “*F01.PB1098*” is the primary transcript. Finally, seven (“*F01.PB10934*”, “*F01.PB1499*”, “*F01.PB3279*”, “*F01.PB1098*”, “*F01.PB4783*”, “*F01.PB1054*”, and “*F01.PB33234*”) genes were identified as core abiotic stress response genes for subsequent analysis. Meanwhile, the annotation information of seven core genes was analyzed (Table S11). These seven DEGs exhibited different levels of response to the four abiotic stress treatments. Interestingly, under four abiotic stress treatments, the expression patterns of these seven genes were consistent in leaves and roots. More specifically, the expressions of “*F01.PB10934*”, “*F01.PB1499*” and “*F01.PB3279*” were down-regulated in both leaf and root tissues. On the contrary, the remaining four genes were significantly up-regulated in leaves and roots than of the control (Fig. 6A and B). In order to further verify the expression patterns of the core stress resistance genes screened in this study in different species, the expression patterns of homologous genes of seven core genes in *M. sativa*, *A. thaliana* and *G. max* under different abiotic stresses were identified through the Blast program. It was found that, compared with the control, they generally responded positively to various stresses and were affected by stress treatment time, but their expression patterns varied greatly in different species, indicating that these seven genes may also play an equally important role in different species (Fig. 6C–P).

Among seven multiple abiotic stress co-responsive genes, “*F01.PB10934*” was annotated as an auxin efflux carrier family protein (PILS7) and has been identified as involved in intracellular auxin transport between the cytosol and endoplasmic reticulum, and in cellular auxin homeostasis by regulating auxin signaling [33]. “*F01.PB1499*” annotation information was dirigent-like (DIR-like) protein, which can be further annotated to DIR19 in *A. thaliana*. It is responsible for stereoselectivity on the phenoxy radical-coupling reaction and play a central role in plant secondary metabolism (including lignans, flavonolignans, and alkaloids biosynthesis), and has been confirmed to be a disease resistance response protein in *Medicago truncatula* [34] The NF-Y transcription factor is composed of three functional subunits: NF-YA, NF-YB and NF-YC, by forming a trimer, recognizing and binding to a CCAAT motif in promoters to stimulate the transcription of a variety of genes [35]. The annotation information of “*F01.PB3279*” was exactly a histone-like TF (CBF/NF-Y), which was highly homologous with nuclear TF Y subunit B-3 (NFYB3) in *A. thaliana*. The annotation result of “*F01.PB1098*” showed that it may be closely related to the late embryogenesis abundant protein group 3 (LEA3). “*F01.PB4783*” participated in the biological process of “defense response” and “response to biotic stimulus” in the annotation of GO database, and its encoded protein abscisic acid (ABA)-responsive protein ABR18 belonged to the pathogenesis-related (PR) protein Bet v 1 family. The encoded protein of “*F01.PB1054*” was classified as dormancy-associated protein 2 (DRM2) in *Pisum sativum*. “*F01.PB33234*” predicted to encode Δ^1^-pyrroline-5-carboxylate dehydrogenase (P5CDh) belonging to the aldehyde dehydrogenases (ALDhs) superfamily. The biological process annotated in this study was “arginine and proline metabolism”, suggesting that it played a role in the conversion of Δ^1^-pyrroline-5-carboxylate (P5C) to glutamate [36].

The PPI networks were constructed by selecting functional proteins homologous to protein sequences encoded by seven core genes from the *A. thaliana* dataset. The following four proteins were queried in *A. thaliana* that matched the input protein sequences, namely AT5G65980, AT1G58170, NF-YB3, and ALDH12A1, corresponding to the gene IDs “*F01.PB10934*”, “*F01.PB1499*”, “*F01.PB3279*”, and “*F01.PB33234*”, respectively (Fig. 4D). Of the remaining three, none exhibited high homology to any known genes in *A. thaliana* and were not retrieved in the STRING database, speculating that these genes may be unique to common vetch. The interactions between proteins formed three modules through the STRING database, including two solitary proteins, i.e., AT5G65980 and AT1G58170; the interactive network between NF-YB3 and STRING-predicted functional protein partners; and the medium confidence interaction network between ALDH12A1 and its functional protein partners.

## 4 Discussion

### 4.1 Comprehensive structural analysis of FL transcripts

The genome sequence contains genetic information related to plant growth and development, biosynthesis of characteristic metabolic components, stress resistance, and other traits. The utilization of genome data of the legume diploid model plant *M. truncatula* is of great significance for accelerating genetic analysis, functional genome and breeding research of *M. truncatula* and other legumes. However, the genomes of most non-model leguminous plants have their own existence value because they contain species specific genes. They may exhibit some unique characteristics and processes in response to abiotic stresses that cannot be approached through model plants. Recently published transcriptome data of some non-model plants have allowed important traits of species to be well revealed at the gene expression level. With the development of current state-of-the-art sequencing technologies, hybrid-sequencing transcriptome analysis followed by identification of candidate genes will help to better understand the molecular mechanism of plant organ formation and development, as well as the stress response. FL transcriptome studies of species based on SMRT sequencing data, supplemented by NGS, have been widely reported, such as *Trifolium ambiguum* [37], *Cassia obtusifolia* [38], and *Linum usitatissimum* [15]. However, the deficiency of information about common vetch in this regard may be one of the factors accounting for the research tends to move along at a particularly slow pace compared with that on other major forage crops. Therefore, in this study, hybrid-sequencing was used to analyze FL transcripts of mixed tissue samples of common vetch under diverse abiotic stress treatments to further explore the sequence information, structural analysis, and functional annotation of FL transcripts.

As described in previous research on other species, our findings confirm the outstanding role of SMRT in capturing longer transcriptional sequences. By comparison, the average length of common vetch transcripts assembled by NGS alone was 1124 bp and an N50 of 1991 bp. Approximately 84.49% of the length of transcripts in SMRT were longer than 1000 bp (Fig. 1), while in NGS, only 55.14% of unigenes met this requirement [39]. Similar results could also be clearly observed in the case of previous studies. In *Camellia sinensis*, about 25.9% of the assembled unigenes from short sequences were less than 500 bp in length, whereas only 2.3% of the isoforms from SMRT were observed [40]. The transcripts directly read from SMRT are longer than assembled transcripts spliced from NGS reads, and it can also be illustrated intuitively by the value of N50 that the transcripts from SMRT are of better quality. On top of that, the results of the sequencing analysis of different tissue samples under multiple abiotic stress treatments were shown to achieve broader transcriptome coverage of the common vetch compared to individual tissue analysis from a single stress treatment or analysis of multiple tissue samples from a single abiotic stress treatment [27]. Due to the abundant sample size of this study, it may provide a more thorough transcriptome of common vetch on the basis of previous studies.

The longer transcriptional sequences also help to improve the accuracy of sequence structure analysis and functional annotation. SSR is a powerful tool with extensive applications in processes such as genetic variation, genetic mapping, and molecular breeding [41]. In this study, a total of 17,227 SSRs were identified, with the highest proportion of mono-nucleotide repeats (7911, 45.92%). In particular, previous studies reported that 454 pyrosequencing technology was used to sequence the transcriptome of common vetch [31], but only 3811 SSR loci were found from 31,504 individuals, less than the 17,227 SSR loci detected in this study, which exposed the defects of NGS short-read length and assembly errors. Alfalfa, which belongs to the same Leguminosae family as common vetch, had more than half of its SSR types be single nucleotide repeats (27,299, 61.99%) [42], and they presumably share similar genomic characteristics. Unlike earlier reports regarding the predominant SSR types in *Rhododendron lapponicum* transcriptional sequences, di-nucleotide repeats (27,639, 42.97%) were the most abundant [10]. The comparison of the findings with those of other studies confirms that SSR abundance generally differs between species, which may be based on species-specific effects and material handling differences. The identification of SSRs in this study provides effective theoretical support for the further development of polymorphic SSR markers in common vetch.

The vast majority of eukaryotic genes and transcripts do not follow a one-to-one correspondence, and a gene usually has a variety of AS events, so that it may hold multiple transcripts simultaneously [43,44]. This ubiquitous process makes the transcripts of the species complex and diverse. Based on the sequence structure analysis of the de-redundant sequences of the mixed samples, we predicted 1059 AS events. Previous researches have confirmed that AS plays a crucial role in plant development, signal transduction, and stress response [45,46]. Therefore, the AS events reported here lay the foundation for further studies on the regulatory mechanisms of gene expression, proteome diversity, and characterization of the functional pathways involved in common vetch.

LncRNAs, defined as a type of RNA transcript longer than 200 nucleotides that cannot be translated into proteins, have attracted increasing attention [47]. In plants, the roles of lncRNAs in flowering regulation, reproductive developmental mediation, and stress response have been experimentally demonstrated [48]. On the contrary, CDSs mainly refer to a class of sequences with very definite protein-coding capability in organisms. In the present study, 28,256 complete CDS regions and 640 candidate lncRNAs were detected. Future experiments on common vetch can focus on these sequences, which will facilitate additional information mining and a better understanding of the role of lncRNA in common vetch. Subsequently, 38,826 transcripts (97.78%) were assigned tentative annotations altogether, among these FL transcripts, the percentage of annotations was higher than that of previous studies on common vetch through NGS (83.48%) [39], and there were also remaining 883 transcripts that were not assigned an annotation by the eight public databases (Table S8), which might represent common vetch specific genes or novel genes or other unknown contaminants, and the majority of which may be involved in resistance to abiotic stress. Furthermore, the results of GO, COG, and KEGG database annotation classification indicated that a great many transcripts held several molecular functions simultaneously and participated in a variety of biological pathways (Table S8), which typified the breadth of gene function. Under the stress of adversity, carbon is preferentially distributed to osmoprotectants, mobilizing the “biosynthesis of amino acids” pathway, and sacrificing the synthesis of storage compounds, such as starch or protein, which is considered a positive manner to cope with abiotic stress. This has been confirmed in previous literature [49]. Strictly controlled the turnover of amino acids and proteins, which is critical to the response of common vetch to abiotic stress. When carbon is deficient, starch can be used as a sugar source for a short time. Under stress, the “starch and sucrose metabolism” pathway is regulated to provide the optimal sugar level required for the stress response. The change in sugar level under stress may be perceived through the stress signal pathway and participate in the stress response through the “plant hormone signal translation” pathway, which affects the growth, development, and production processes of plants under adverse conditions.

In a nutshell, our sequencing results provided almost comprehensive data on FL transcripts in common vetch, which greatly enriched the transcriptome database for common vetch and elucidated the structural and functional characterization of transcripts.

### 4.2 Regulation of TFs on common vetch resistance to stress

TFs are key regulatory proteins necessary for regulating gene expression. TFs can activate or inhibit the transcription of multiple target genes by combining with specific motifs in target genes in living organisms [37], so as to guide the growth, development, and metabolic processes of multifarious plants and also fulfill a vital role in signal transduction and promote the tolerance or adaptation of plants to adverse environments [50,51]. In the present study, among the 64 TF families identified, C3H, WRKY, and bHLH were listed as the top three TF families in common vetch.

C3H proteins are widely involved in regulating plant developmental processes and stress adaptation [52,53]. For example, in *A. thaliana*, C3H zinc finger protein AtZFP1 tended to endow plants with salt tolerance by regulating ion balance, relieving osmotic equilibrium, and maintaining reactive oxygen species (ROS) homeostasis [54]. The WRKY TF family is a large gene superfamily of the plant genome, which is an integral part of the regulatory network regulating the stress response process of varied plants [55]. Extensive evidences have been demonstrated that WRKY proteins specifically recognize and bind to the *cis*-acting element called the W-box (TTGACC/T) in the promoters of downstream target genes [56,57]. *PbrWRKY53* isolated from *Pyrus betulaefolia* directly interacted with a W-box element on the promoter of *PbrNCED1* to regulate the expression of this gene, thus playing a positive regulatory role in mediating drought resistance [55]. In another case, in *A. thaliana*, VQ9 and WRKY8 play an antagonistic role. VQ9 protein proved to be a repressor of WRKY8 factor, maintains the balance of the WRKY8-mediated signal pathway through protein–protein interactions, and establishes salinity stress tolerance [58]. The bHLH TF family is defined by its highly conserved bHLH signature domain [50,59]. The bHLH TF AhHLH112 in *Arachis hypogaea* mediated H_2_O_2_ homeostasis by enhancing the activity of antioxidant enzymes and scavenging ROS produced during stress, and may participate in ABA-dependent stress response pathway to enhance drought stress tolerance [59].

Therefore, a large number of TFs enriched in this study may activate a host of target genes with specific domains, which play a synergistic anti-stress role or mediate abiotic stress responses by participating in various signal pathways. Another possibility is that there may be proteins interacting with these TFs proteins in common vetch, which can mitigate the adverse effects caused by abiotic stress.

### 4.3 Prediction of stress-related candidate genes

Since the expression of specific genes in plants undergoes different types of changes under different abiotic stress treatments, it is reasonable to predict that these genes can respond to corresponding abiotic signals [3]. Transcriptome analysis has greatly promoted the discovery of candidate functional genes or proteins related to various abiotic stress treatments [60]. Considering that the molecular mechanisms of plant tolerance to abiotic stress are not independent, often simultaneously exist under natural conditions [15,61]. Here, we employed a hybrid-sequencing strategy to analyze gene co-expression data under three single stresses (NaCl, drought, cold) and one stress combination (cold drought) in both aboveground leaves and underground roots of common vetch and showed specific examples of the utility of the gene expression atlas in the multiple abiotic stress responsive candidate genes identification. Based on the significant up- and down-regulation of gene expression, seven leaf-root co-responsive candidate genes were found to be involved in the responses of the above all four stress treatments, indicating that these genes might play a regulatory role in resisting multiple abiotic stresses in common vetch [61].

Three down-regulated genes and one up-regulated gene “*F01.PB33234*” under the above four stress treatments were matched with highly homologous expression proteins in *A. thaliana* through the STRING. By tracking all available types of protein association evidence and prediction algorithms, assembling all known and predicted protein functional associations in STRING can provide us with basic information about the specific biological function or pathway in which paired proteins are jointly involved [62,63]. Most studies focused on the up-regulated genes, which were predicted to be stress resistant genes. However, genes down-regulated during abiotic stress could also prove a decisive factor in stress resistance [64].

“*F01.PB10934*” was highly homologous to *PILS7*, which encoded auxin efflux carrier family protein. It is a key gene regulating the complex mechanism of development and growth in plants, involving somatic embryogenesis [33], but has not been shown to play a role in abiotic defense responses. Further dissection of whether this gene can endow common vetch with abiotic tolerance will likely require relying on molecular biology technology to verify whether there is a change in abiotic resistance. An *A. thaliana* DIR-like family protein was highly homologous to the protein encoded by “*F01.PB1499*”. Recently, researchers have outlined the role of DIR-like proteins in biotic and abiotic stress responses. Lignans biosynthesis pathway contributes significantly to defense against pathogens [65]. In addition, the expression of several of the DIR-like genes accompanied by the regulation of specific peroxidases were responsive to temperature and cold stresses, but the expression patterns were almost different [65]. Previous study from *Saccharum* spp. reported that *ScDir* gene was involved in the response to abiotic stresses such as drought, salt, and oxidant [66]. The phylogenetic tree of the expanded gene family of DIR and DIR-like proteins suggests that there may be multiple distinct subfamilies [34]. Each subfamily contains many genes with relatively high sequence differences, some of which are adjacent to each other or form an independent branch of phylogenetic tree subfamily, possibly indicating that individual genes have different functional roles and expression patterns [67]. Similarly, “*F01.PB1499*” is also likely to be a candidate gene for stress response in common vetch, but further research efforts are still needed to determine the definitive mechanisms underpinning common vetch to cope with the diverse abiotic stress. “*F01.PB3279*” matched the homologous gene coding NF-YB3 protein in *A. thaliana*. The wide diversity of plant NF-Y proteins increases the possibility of different combinations of aforementioned three subunits to produce trimers with different molecular functions. The strong interaction between *NF-Y* family gene coding proteins can also be seen from PPI network (Fig. 4D). From the previous research data of *A. thaliana*, it could be concluded that NF-YB3 had a negative effect on the induction of dehydration induced genes during dehydration stress, but it had a positive effect on the induction of heat-inducible genes under heat stress [68]. *NF-Y* gene family plays distinguished roles in plant abiotic stress tolerance but has functionally diverged and expression patterns differentiated in different species. Therefore, the molecular mechanism of NF-YB3 in different stress of common vetch needs to be further prospected.

LEA3 proteins are found widespread in various organisms and are considered to be important elements that improve the tolerance of plants to various abiotic stress like drought or salinity [69]. Among the following five up-regulated genes, which were also the most likely candidate genes for stress-related. “*F01.PB1098*” was annotated in the database as *LEA3*, which were much higher expressions than the other six genes under various stress treatments in common vetch. This finding was consistent with the previous research, the molecular and functional characteristics of *Gh_A08G0694* which belonged to the LEA3 were found to be strongly induced in leaves and roots during drought and salt stress in *Gossypium hirsutum*, the knockout of *GhLEA3* (*Gh_A08G0694*) gene improved the sensitivity of cotton to drought and salt stress, while overexpression enhanced the resistance to drought and salt stress [70]. The high homology in protein sequences and the similar gene expression patterns imply that they may have the same function. Therefore, we predicted that the two genes screened in common vetch also could play the same role in enhancing abiotic stress tolerance. *PR* genes are the main defense genes induced in response to pathogen attack, they also regulate the expression of other proteins that prevent pathogens from entering the site of infection [71]. The encoded protein of “*F01.PB4783*” belonged to the PR Bet v 1 family. Bet v 1 is homologous to a large number of PR proteins (PRs). PRs accumulate in plants as part of resistance to pathogen attack [72]. According to the structure, phylogeny and biological activity, a large number of identified PRs are divided into 17 functional families. Among them, PR-1 is the most produced and studied protein in a variety of abiotic stress defense responses [71,73]. The different types of changes in the expression of *BrBetv1AFP* in *Brassica rapa* under abiotic stress treatments suggest that it may be related to abiotic stress resistance [74]. However, to the best of our knowledge, the functional analysis of the *PR Bet v 1* family to confirm its abiotic resistance in common vetch is almost blank, and we hope that this gene can receive attention in future species resistance gene studies. The common vetch “*F01.PB1054*” gene had been annotated as DRM2, and it was a negative regulator of basal defense against highly pathogenic bacteria. The positive and negative regulators form a hyperfine structure and are triggered to fine-tune defensive responses, which can be clearly found in *A. thaliana*, both *DRM2* and *flowering locus D* (*FLD*) gene were up-regulated after systemic weakened resistance activation, predicted that the co-upregulation of *DRM2* and *FLD* gene may be a process of fine-tuning the output of plant defense response [75]. It is reasonable to infer that there may also be a delicate structure co-expressed with “*F01.PB1054*” that conferred significant advantages in response to abiotic stress in common vetch. The massive accumulation of proline (Pro) is a common physiological response of many organisms under a wide range of abiotic stresses. However, the accumulation of Pro in plants does not cause damage to cells per se, but is caused by P5C, an intermediate product of Pro biosynthesis and degradation [36]. “*F01.PB33234*” was expected to encode mitochondrial P5CDH, which played a key role in maintaining redox homeostasis, protecting cells from Pro toxicity, and promoting cell growth and immunomodulation by degrading P5C. Under various stress treatments, the increased expression of *P5CDh* in *Litopenaeus vannamei*, suggested that after removing P5C and converting it to glutamate, the enzyme played a protective role by reducing the production of reactive oxygen species [76]. Likewise, the up-regulation of gene in this study may be that common vetch itself perhaps in anticipation of the proline accumulation, which we speculate that this may be a protecting mechanism in common vetch during various abiotic stress treatments. Given all of that, transcriptome sequencing data, public database annotation of transcripts and PPI networks provided preliminary evidence support for the seven candidate genes identified.

## 5 Conclusion

Overall, as expected, we identified seven candidate genes underlying responses to stresses associated with common vetch by gene expression data, annotation information and PPI networks. This study provided a valuable sequence resource and deepened our understanding of the FL transcripts of common vetch. Based on this result, screening and identifying candidate genes related to multiple abiotic stresses will play a valuable role in common vetch breeding.

## Author contribution statement

Jia Wei: Performed the experiments; Analyzed and interpreted the data; Wrote the paper.

Bo Luo, Shiyi Kong: Performed the experiments; Analyzed and interpreted the data.

Wenxian Liu, Zhenwu Wei: Conceived and designed the experiments; Contributed reagents, materials, analysis tools or data.

Chuanjie Zhang: Contributed reagents, materials, analysis tools or data.

Xueyang Min: Conceived and designed the experiments; Contributed reagents, materials, analysis tools or data; Wrote the paper.

## Funding statement

Dr Xueyang Min was supported by the 10.13039/501100010023Natural Science Foundation of the Jiangsu Higher Education Institutions of China [22KJB230011], 10.13039/501100004608Natural Science Foundation of Jiangsu Province [BK20220583], Jiangsu Planned Projects for Postdoctoral Research Funds [2021K197B].

Zhenwu Wei was supported by Shanghai Agriculture Applied Technology Development Program [No. T20200102].

## Data availability statement

Data included in article/supp. material/referenced in article.

## Declaration of interest’s statement

The authors declare that they have no known competing financial interests or personal relationships that could have appeared to influence the work reported in this paper.

## Appendix A Supplementary data

Multimedia component 11 Multimedia component 11

figs1 Venn diagram of *Vicia sativa* lncRNAs predicted by CPC, CNCI, CPAT and Pfam methods. Overlap displayed the number of shared detected lncRNAs. figs1

figs2 Histogram of GO terms for all annotated transcripts in biological process, cellular component and molecular function. The X-axis showed GO categories, the left Y-axis indicated the sequence percentage of each category, and the right Y-axis represented the total number of transcripts in each category. figs2

figs3 COG function annotation classification statistics of *Vicia sativa* transcripts. The X-axis represented the function classes, and the Y-axis indicated the frequency of transcripts in each specific functional cluster. figs3
