# Supplementary material for: Screening and identification of multiple abiotic stress responsive candidate genes based on hybrid-sequencing in Vicia sativa
Source: Heliyon. 2023 Feb 4;9(2):e13536. doi: 10.1016/j.heliyon.2023.e13536 (PMC9929474; doi:10.1016/j.heliyon.2023.e13536)
Supplement: Multimedia component 1 [file mmc1.docx]

Table S1 Summary of polymerase reads from SMRT sequencing.

| cDNA size | 1-2K | 2-3K | 3-6K | All |
| --- | --- | --- | --- | --- |
| SMRT cells | 3 | 2 | 2 | 7 |
| Polymerase reads | 450,876 | 300,584 | 300,584 | 1,052,044 |
| Post-filter polymerase reads | 277,963 | 216,823 | 231,665 | 726,451 |
| Post-filter total number of subread bases | 6,776,596,893 | 5,613,255,715 | 5,555,961,896 | 17,945,814,504 |
| Post-filter number of subread | 5,249,161 | 2,185,332 | 1,336,968 | 8,771,461 |
| Post-filter subreads N50 | 1,259 | 2,583 | 4,157 | 2,666 |
| Post-filter mean subread length | 1,290 | 2,568 | 4,155 | 2,045 |
