# Supplementary material for: Screening and identification of multiple abiotic stress responsive candidate genes based on hybrid-sequencing in Vicia sativa
Source: Heliyon. 2023 Feb 4;9(2):e13536. doi: 10.1016/j.heliyon.2023.e13536 (PMC9929474; doi:10.1016/j.heliyon.2023.e13536)
Supplement: Multimedia component 5 [file mmc5.docx]

Table S5 Summary of predicted target mRNA transcripts corresponding to each lncRNA.

| LncRNA ID | Target mRNA ID |
| --- | --- |
| F01.PB10426 | F01.PB20297 |
| F01.PB11332 | F01.PB12643 |
| F01.PB12212 | F01.PB35242 |
| F01.PB12542 | F01.PB37317; F01.PB37250; F01.PB12981 |
| F01.PB1340 | F01.PB20206; F01.PB34932; F01.PB37636 |
| F01.PB1443 | F01.PB38228; F01.PB22963 |
| F01.PB1620 | F01.PB12643 |
| F01.PB1705 | F01.PB38228; F01.PB22963 |
| F01.PB1716 | F01.PB14715; F01.PB11194; F01.PB36903 |
| F01.PB17296 | F01.PB19067; F01.PB21914 |
| F01.PB1751 | F01.PB22934 |
| F01.PB18760 | F01.PB9247; F01.PB20390 |
| F01.PB1879 | F01.PB23728; F01.PB39431; F01.PB39612 |
| F01.PB1956 | F01.PB10383 |
| F01.PB2 | F01.PB32224; F01.PB32251; F01.PB32245 |
| F01.PB20088 | F01.PB31668 |
| F01.PB26740 | F01.PB24882; F01.PB26131; F01.PB25568 |
| F01.PB29140 | F01.PB24248; F01.PB16589; F01.PB33734 |
| F01.PB30240 | F01.PB18368; F01.PB13621 |
| F01.PB30304 | F01.PB9023; F01.PB1230 |
| F01.PB30494 | F01.PB37807; F01.PB25505; F01.PB27699 |
| F01.PB30752 | F01.PB8921 |
| F01.PB30807 | F01.PB21111; F01.PB13646; F01.PB11842 |
| F01.PB30916 | F01.PB34196; F01.PB32251; F01.PB32224 |
| F01.PB30994 | F01.PB39579; F01.PB34076; F01.PB34448 |
| F01.PB31018 | F01.PB9020; F01.PB8322; F01.PB30908 |
| F01.PB31081 | F01.PB4568 |
| F01.PB31248 | F01.PB32148 |
| F01.PB31349 | F01.PB39189 |
| F01.PB32152 | F01.PB34347; F01.PB39257; F01.PB39181 |
| F01.PB32171 | F01.PB34058; F01.PB1098; F01.PB34090 |
| F01.PB32191 | F01.PB22085; F01.PB15083; F01.PB33123 |
| F01.PB32235 | F01.PB39223; F01.PB34177; F01.PB32213 |
| F01.PB32242 | F01.PB5447 |
| F01.PB32290 | F01.PB814; F01.PB34272; F01.PB32199 |
| F01.PB32803 | F01.PB38692 |
| F01.PB3311 | F01.PB34249 |
| F01.PB33405 | F01.PB34578 |
| F01.PB33483 | F01.PB11912; F01.PB34419; F01.PB31019 |
| F01.PB3365 | F01.PB18848 |
| F01.PB33821 | F01.PB38692 |
| F01.PB33838 | F01.PB10234 |
| F01.PB3405 | F01.PB32178 |
| F01.PB34199 | F01.PB2877 |
| F01.PB34210 | F01.PB5880 |
| F01.PB34290 | F01.PB30279 |
| F01.PB34302 | F01.PB39444 |
| F01.PB34440 | F01.PB26695; F01.PB24212; F01.PB31077 |
| F01.PB34477 | F01.PB39368 |
| F01.PB34581 | F01.PB8459 |
| F01.PB3591 | F01.PB39076 |
| F01.PB37388 | F01.PB24475; F01.PB19964; F01.PB20223 |
| F01.PB38295 | F01.PB30274; F01.PB3347; F01.PB30265 |
| F01.PB39260 | F01.PB4253 |
| F01.PB39292 | F01.PB34196; F01.PB32251; F01.PB32245 |
| F01.PB3979 | F01.PB34361 |
| F01.PB4205 | F01.PB34249 |
| F01.PB4473 | F01.PB7931 |
| F01.PB4711 | F01.PB32144 |
| F01.PB5006 | F01.PB33812 |
| F01.PB5086 | F01.PB19000 |
| F01.PB5135 | F01.PB10827 |
| F01.PB5156 | F01.PB11597 |
| F01.PB5173 | F01.PB10383 |
| F01.PB5524 | F01.PB39196; F01.PB39160; F01.PB34452 |
| F01.PB554 | F01.PB32829 |
| F01.PB8068 | F01.PB789 |
| F01.PB8196 | F01.PB39675 |
| F01.PB849 | F01.PB22792; F01.PB27967; F01.PB26218 |
| F01.PB8999 | F01.PB13179 |
| F01.PB955 | F01.PB20780 |
| F01.PB9819 | F01.PB6187 |
| F01.PB9831 | F01.PB16054 |
