# Supplementary material for: Screening and identification of multiple abiotic stress responsive candidate genes based on hybrid-sequencing in Vicia sativa
Source: Heliyon. 2023 Feb 4;9(2):e13536. doi: 10.1016/j.heliyon.2023.e13536 (PMC9929474; doi:10.1016/j.heliyon.2023.e13536)
Supplement: Multimedia component 7 [file mmc7.docx]

Table S7 TFs prediction data.

| Family | Number | Percentage |
| --- | --- | --- |
| C3H | 146 | 8.86% |
| WRKY | 94 | 5.71% |
| bHLH | 92 | 5.59% |
| bZIP | 85 | 5.16% |
| MYB-related | 85 | 5.16% |
| C2H2 | 82 | 4.98% |
| GRAS | 76 | 4.61% |
| AP2/ERF-ERF | 75 | 4.55% |
| MYB | 68 | 4.13% |
| NAC | 55 | 3.34% |
| B3-ARF | 51 | 3.10% |
| SBP | 48 | 2.91% |
| HB-HD-ZIP | 46 | 2.79% |
| B3 | 42 | 2.55% |
| Trihelix | 39 | 2.37% |
| FAR1 | 36 | 2.19% |
| GARP-G2-like | 33 | 2.00% |
| CAMTA | 29 | 1.76% |
| TUB | 29 | 1.76% |
| C2C2-Dof | 27 | 1.64% |
| C2C2-GATA | 23 | 1.40% |
| HB-other | 21 | 1.28% |
| Zn-clus | 21 | 1.28% |
| Alfin-like | 20 | 1.21% |
| TCP | 20 | 1.21% |
| HB-BELL | 19 | 1.15% |
| Tify | 19 | 1.15% |
| Family | Number | Percentage |
| HSF | 17 | 1.03% |
| GARP-ARR-B | 15 | 0.91% |
| RWP-RK | 14 | 0.85% |
| NF-YB | 13 | 0.79% |
| BES1 | 12 | 0.73% |
| LOB | 12 | 0.73% |
| NF-YC | 11 | 0.67% |
| AP2/ERF-AP2 | 10 | 0.61% |
| C2C2-CO-like | 10 | 0.61% |
| EIL | 10 | 0.61% |
| ZF-HD | 10 | 0.61% |
| MADS-M-type | 9 | 0.55% |
| NF-X1 | 9 | 0.55% |
| VOZ | 9 | 0.55% |
| C2C2-YABBY | 8 | 0.49% |
| MADS-MIKC | 8 | 0.49% |
| NF-YA | 8 | 0.49% |
| HB-KNOX | 7 | 0.43% |
| C2C2-LSD | 6 | 0.36% |
| OFP | 6 | 0.36% |
| PLATZ | 6 | 0.36% |
| DBB | 5 | 0.30% |
| E2F-DP | 5 | 0.30% |
| GeBP | 5 | 0.30% |
| HB-WOX | 5 | 0.30% |
| SRS | 5 | 0.30% |
| Whirly | 5 | 0.30% |
| BBR-BPC | 4 | 0.24% |
| CPP | 4 | 0.24% |
| LIM | 4 | 0.24% |
| CSD | 3 | 0.18% |
| AP2/ERF-RAV | 2 | 0.12% |
| GRF | 2 | 0.12% |
| HB-PHD | 2 | 0.12% |
| STAT | 2 | 0.12% |
| ULT | 2 | 0.12% |
| DBP | 1 | 0.06% |
