# Supplementary figures and images for: Screening and identification of multiple abiotic stress responsive candidate genes based on hybrid-sequencing in Vicia sativa
Source: Heliyon. 2023 Feb 4;9(2):e13536. doi: 10.1016/j.heliyon.2023.e13536 (PMC9929474; doi:10.1016/j.heliyon.2023.e13536)

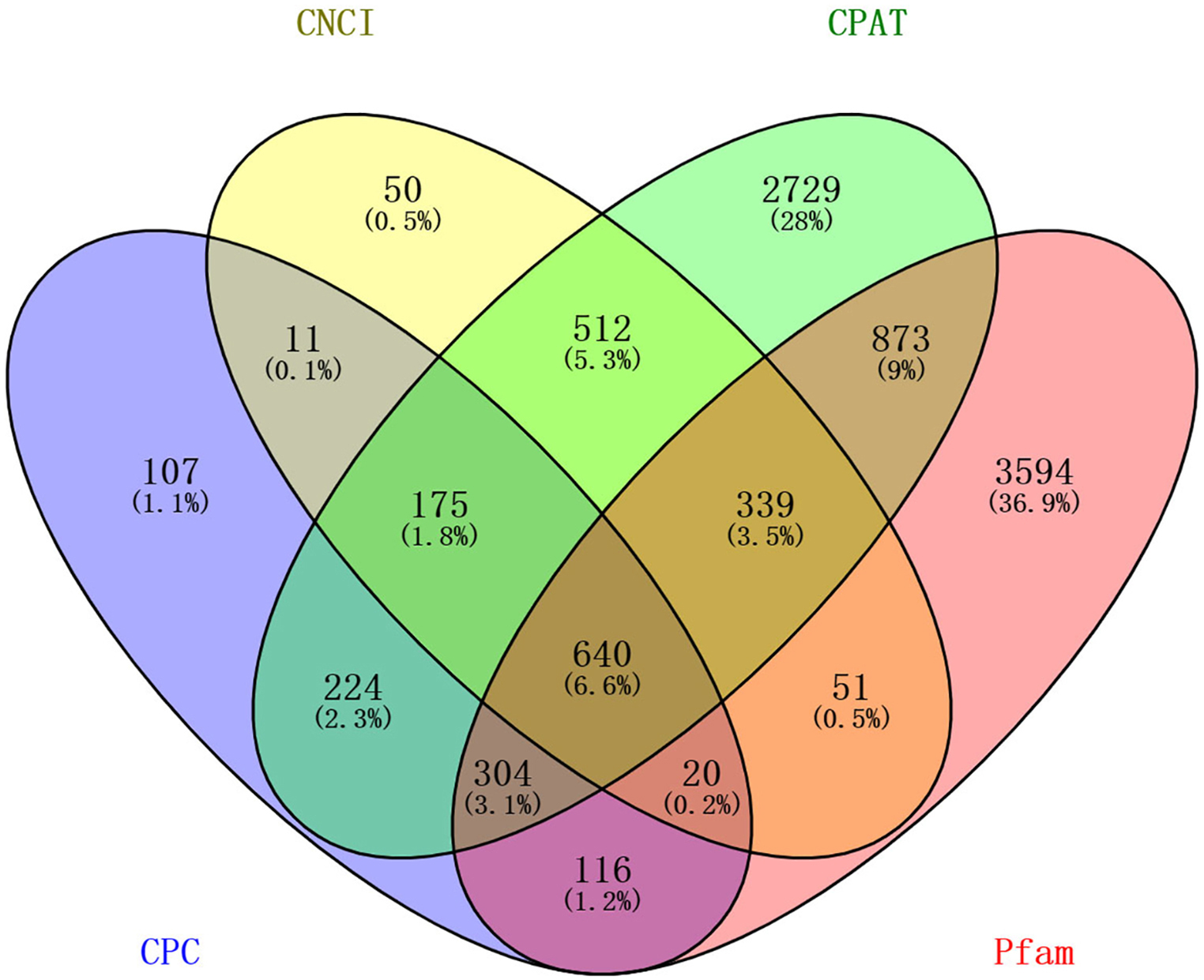

Supplement: figs1 — Venn diagram of Vicia sativa lncRNAs predicted by CPC, CNCI, CPAT and Pfam methods. Overlap displayed the number of shared detected lncRNAs. [file mmcfigs1.jpg]

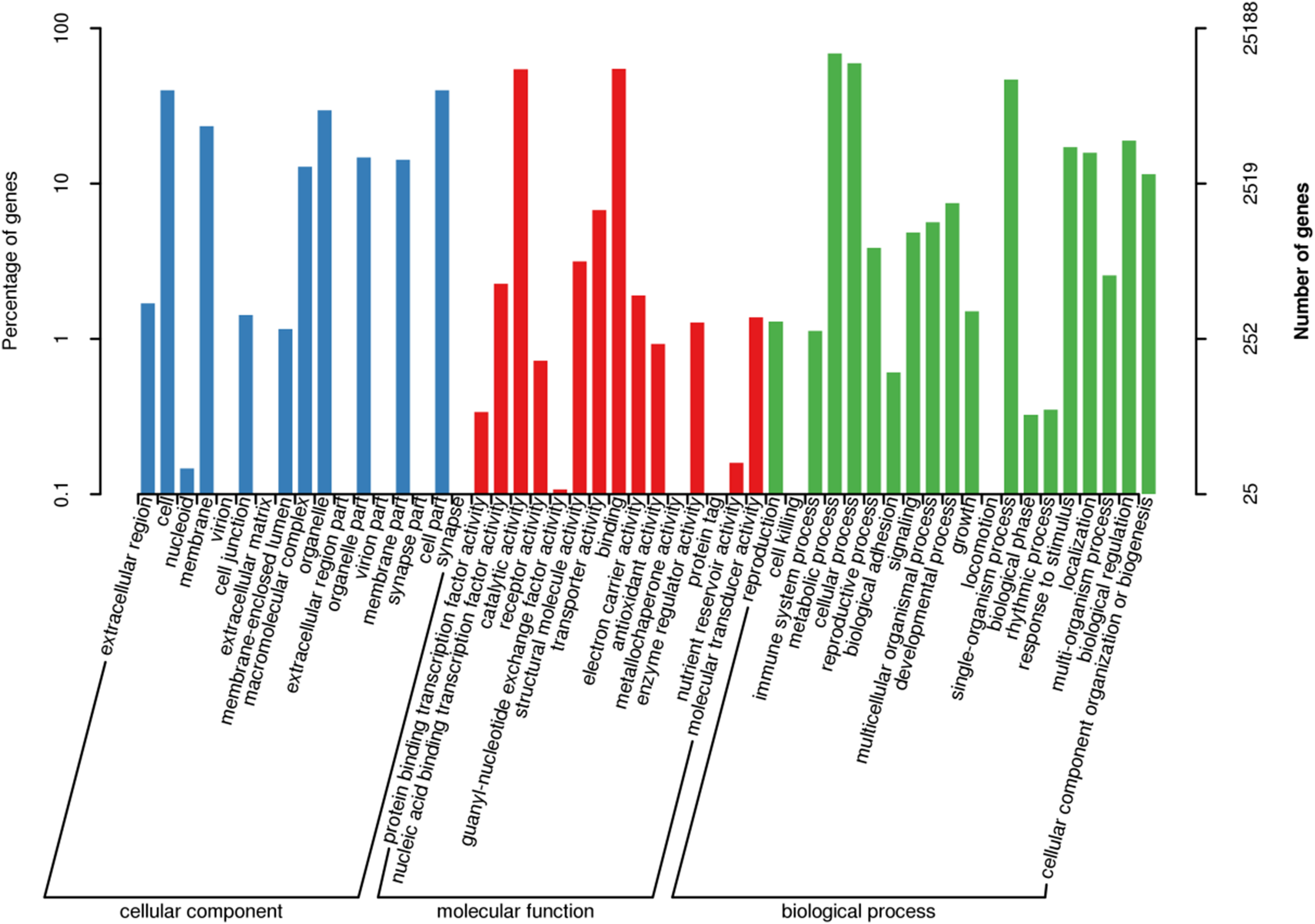

Supplement: figs2 — Histogram of GO terms for all annotated transcripts in biological process, cellular component and molecular function. The X-axis showed GO categories, the left Y-axis indicated the sequence percentage of each category, and the right Y-axis represented the total number of transcripts in each category. [file mmcfigs2.jpg]

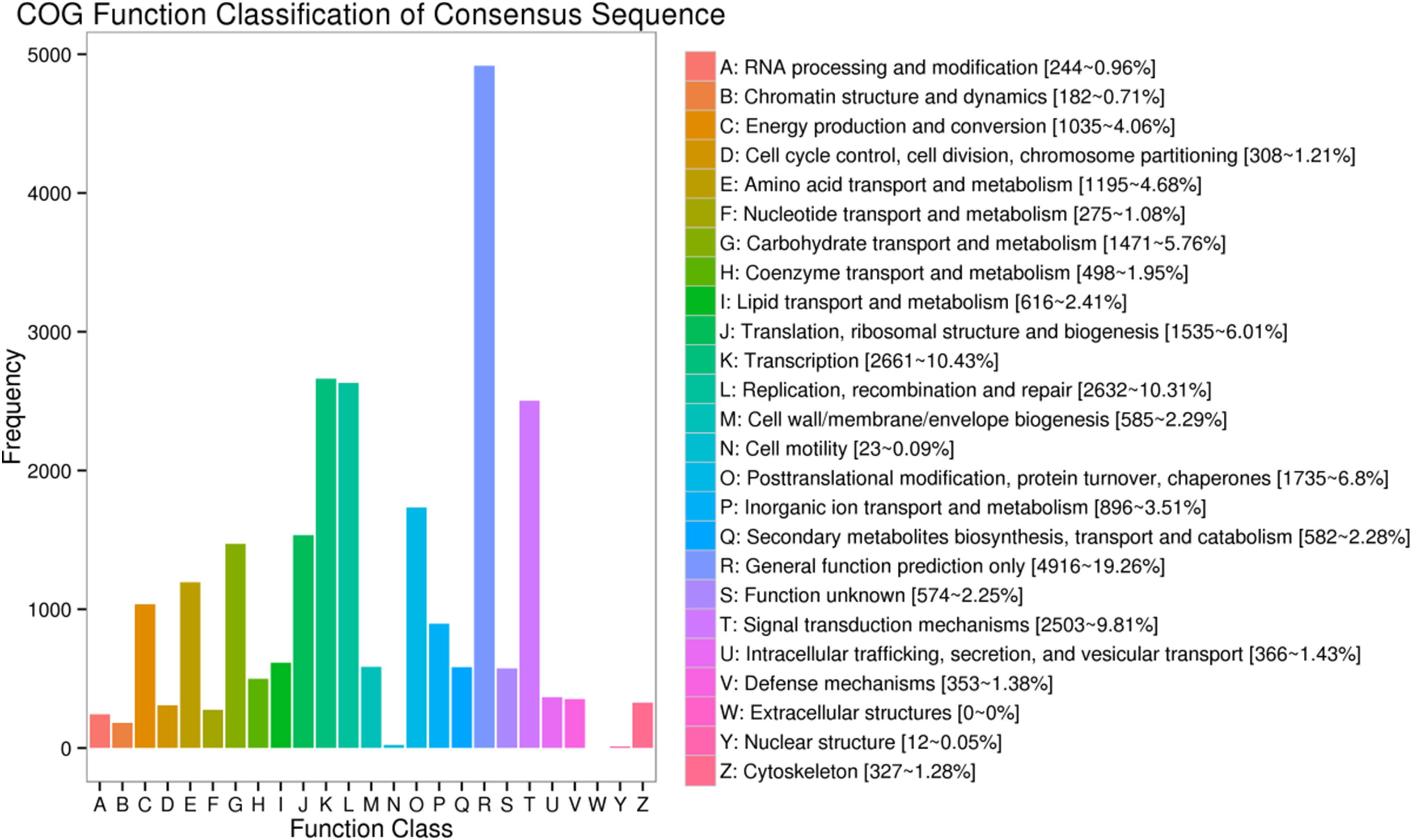

Supplement: figs3 — COG function annotation classification statistics of Vicia sativa transcripts. The X-axis represented the function classes, and the Y-axis indicated the frequency of transcripts in each specific functional cluster. [file mmcfigs3.jpg]
